# Unraveling Ultrafast Li-Ion Dynamics in the Solid Electrolyte LiTi_2_(PS_4_)_3_ by NMR down to Cryogenic Temperatures

**DOI:** 10.1021/jacs.5c05253

**Published:** 2025-06-02

**Authors:** Denise Tapler, Bernhard Gadermaier, Jonas Spychala, Florian Stainer, Annika Marko, Jana Königsreiter, Katharina Hogrefe, Paul Heitjans, H. Martin R. Wilkening

**Affiliations:** † Institute of Chemistry and Technology of Materials (NAWI Graz), 27253 Graz University of Technology, Stremayrgasse 9, 8010 Graz, Austria; ‡ Institute of Physical Chemistry and Electrochemistry, 26555 Leibniz Universität Hannover, Callinstraße 3-3a, 30167 Hannover, Germany

## Abstract

Self-diffusion processes of small atoms or ions play a crucial role in many areas of research. The unique crystal structure of LiTi_2_(PS_4_)_3_ (LTPS) presents a variety of energetically inequivalent diffusion pathways for small Li^+^ charge carriers and has resulted in one of the highest Li^+^ diffusion coefficients. Investigating these pathways individually at the atomic scale poses significant challenges, especially for probing jump processes. In this study, we utilized nuclear spin relaxation techniques down to cryogenic temperatures (10 K) to reveal unprecedented details about both long-range and short-range Li^+^ dynamics. The temperature-dependent ^7^Li NMR spin–lattice relaxation (SLR) rate exhibits a series of diffusion-induced peaks, allowing the extraction of activation energies and jump rates. Due to the exceptionally fast localized Li^+^ exchange processes in LTPS, temperatures as low as 50 K are required to freeze Li^+^ dynamics, on the SLR time scale, entirely within the ring-like cages of the LTPS structure.

## Graphical abstract

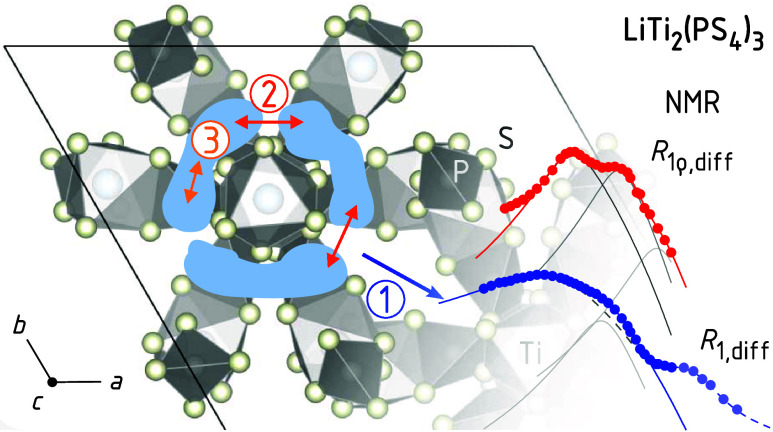

## 1 Introduction

The rising levels of anthropogenic CO_2_ and other greenhouse gases compel us to transition to energy systems based on so-called “renewable sources”. The intermittent nature of renewable energy, such as wind, tidal, and solar power, necessitates efficient energy storage solutions to balance supply and demand. Among these, electrochemical devices hold a leading position due to their high energy efficiency and adaptability for various applications.^1–3^

To meet the growing demand for high-energy and high-power rechargeable batteries—critical not only for large-scale stationary energy storage^4^ but also for electric vehicles^5^ —substantial efforts have been devoted to enhancing the capacity, power, safety, and lifespan of conventional^6–16^ and all-solid-state Li-based batteries.^17–21^ While significant advances have been made in systems utilizing liquid organic electrolytes,^22–27^ all-solid-state batteries (ASSBs) that incorporate ceramic solid electrolytes^28–37^ have emerged as a particularly promising next-generation technology.^17,20,37–39^ These systems offer notable advantages, including enhanced stability, energy density, and power.^37,40^ Replacing highly flammable liquid electrolytes with solid electrolytes mitigates safety risks such as inflammability and leakage, and, it is hoped, also improves the cycle life of such systems.^37^ The development of such systems is, however, only possible with materials that offer electrochemically stable interfaces.^20,41–47^ If available, such electrolytes would also enable the use of high-capacity electrode materials, such as metallic lithium as the negative electrode,^20^ resulting in higher energy densities. Furthermore, ceramic solid electrolytes are considered promising for next-generation battery systems like Li–O_2_ and Li–S batteries,^48–52^ which offer even greater capacity potential. Another key advantage of ASSBs is their ability to operate at elevated temperatures, making them suitable for a larger range of electrochemical applications.^32,53^

For a solid to be deemed a suitable electrolyte in ASSBs, it must exhibit a very high lithium-ion conductivity at ambient temperature, ideally comparable to or even surpassing the ionic conductivity of conventional liquid electrolytes.^40,54–57^ Consequently, the search for materials with fast ion dynamics and negligible electronic conductivity has propelled the development of “superionic” conductors to unprecedented levels.^30,36,58–61^ In recent years, several highly conductive crystalline families, including oxides and sulfides such as NASICON-type structures,^32,62–64^ perovskites,^65–68^ garnets,^58–60,69–73^ argyrodites,^35–37,74–76^ and other thiophosphates such as Li_7_P_3_S_11_,^77–79^ and Li_10_GeP_2_S_12_ (LGPS),^80–82^ have been identified and studied. Recently, also oxyhalides^83–88^ and high-entropy materials^89–94^ entered the spotlight of research.

Among these different materials, LiTi_2_(PS_4_)_3_ (LTPS) has garnered significant attention for exhibiting a very high Li-ion diffusion coefficient being on par with those of the LGPS-type materials.^55^ Structural analyses, pulsed-field gradient (PFG) nuclear magnetic resonance (NMR), impedance spectroscopy, and ab initio molecular dynamics simulations (AIMD) have provided valuable insights into the “liquid-like” diffusion of lithium ions in this compound.^55^ However, detailed knowledge about the elementary steps governing Li^+^ hopping at shorter length scales remains limited — yet these are critical for understanding bulk ion transport and the conductivity properties of this intriguing solid electrolyte. LTPS exhibits unique structural motifs that enable length-scale-dependent ion dynamics, ranging from spatially restricted hopping to extended transport. As such, it is not only highly relevant for battery applications but also serves as a model system for fundamental spectroscopic studies.

In this study, we employ ^7^Li spin–lattice relaxation (SLR) NMR experiments^95^ to measure diffusion-induced relaxation rates in LTPS over a wide temperature range.^96^ In general, time-domain NMR spectroscopy, particularly diffusion-controlled relaxation and line-shape measurements, offers a powerful, contactless, and nondestructive method to explore Li^+^ dynamics across various length and time scales.^95–99^ This technique enables the determination of key dynamic parameters, including activation energies, jump rates, and diffusion coefficients.^74,100–103^ By analyzing the diffusion-induced ^7^Li NMR rate peaks of LTPS, we deduce activation energies and Li^+^ jump rates. Moreover, NMR line-shape measurements^95,102^ provide additional insights into Li^+^ dynamics within LTPS with its various diffusion pathways. The findings are compared with results from PFG NMR, impedance spectroscopy, and AIMD simulations,^55^ offering a further step toward a comprehensive view on Li^+^ dynamic processes in LiTi_2_(PS_4_)_3_.

## 2 Experiment

### 2.1 Synthesis and Structural Characterization

The LTPS sample used for the NMR measurements in this study was previously prepared and structurally characterized elsewhere.^55^ For this work, we utilized the same sample, which was fire-sealed in glass ampoules under vacuum. This procedure preserves the samples and protects them from reactions with moisture over time.

### 2.2 NMR Measurements

As mentioned, due to their high sensitivity to moisture and air, the pressed pellets (obtained from Toyota) were fire-sealed in glass tubes prior to the NMR measurements. ^7^Li NMR SLR rates, both in the laboratory frame and the rotating frame of reference, were recorded using a Bruker 300 Avance spectrometer coupled with a shimmed cryomagnet (*B*_0_ = 7 T), which operated at a Larmor frequency of ω_0_/2π = 116.4 MHz. A high-temperature ceramic NMR probe head (Bruker Biospin) was used for temperature measurements ranging from 175 to 533 K, in conjunction with an Eurotherm unit and a type T thermocouple for temperature control and monitoring. This broadband probe allowed π/2 pulse lengths of approximately 2.5 μs at 200 W. For temperatures as low as 10.2 K, a cryoprobe from Bruker, operated with evaporated liquid nitrogen and helium, was employed. Temperature adjustments and monitoring were carried out using a LakeShore 331 unit, combined with two Cernox sensors. This setup^96^ enabled us to work at temperatures as low as 10 K under rather stable conditions (±0.2 K) for more than 12 h. At 90 W, the π/2 pulse lengths ranged from 3.5 to 6.5 μs depending on the temperature in the sample chamber.

To measure the ^7^Li NMR SLR rates (*R*_1_ and *R*_1ρ_), we used the well-known saturation recovery pulse sequence and the spin-lock technique.^104^ The saturation recovery pulse sequence consisted of a train of ten 90° pulses (each 2.5 μs in length) that disrupted any longitudinal magnetization; its recovery as a function of waiting time was then recorded until full recovery was achieved. The magnetization transients were analyzed with stretched exponentials to extract the diffusion-induced SLR rates *R*_1_. The stretching exponents ranged from 1 to 0.8, showing only slight deviations from simple exponential recovery. In general, for transients from spin-3/2 nuclei exposed to sufficiently large quadrupole interactions biexponential behavior^105^ might be observed,^106,107^ see Supporting Information. For the spin-lock measurements, we used a locking frequency of ω_1_/2π = 20 kHz and locking pulse durations between 22 μs and 460 ms. The recycle delay was set to 5 × 1/*R*_1_ to ensure full longitudinal recovery. The corresponding rates *R*_1ρ_ were obtained by evaluating the spin-lock transversal magnetization transients with stretched exponentials; stretching exponents ranged from 0.45 to 0.9 depending on the temperature. Static ^7^Li NMR line shapes were recorded after exciting the spin ensemble with a single 90° pulse. Again, the recycle delay was set to at least 5 × 1/*R*_1_ to ensure quantitative line shapes, which were obtained after Fourier transformation of the free induction decays.

## 3 Results and Discussion

Figure 1a shows the temperature-evolution of the ^7^Li NMR SLR rates *R*_1_ (ω_0_/2π = 116 MHz) and *R*_1ρ_ (ω_1_/2π = 20 kHz) of LTPS using an Arrhenius diagram. When coming from high temperatures *T*, *R*_1_ (see the left axis) passes through a rather broad rate peak (B) that is located at *T*_max,B_ = 343 K. With decreasing *T*, another rate peak (A) is passed through A (*T*_max,A_ = 214 K). The latter appears as a shoulder of peak B, before the rate enters a temperature regime where it seems to depend only weakly on temperature. The diffusion-controlled peaks A and B are also seen when we turn to the ^7^Li NMR rates *R*_1ρ_ rates. The corresponding peaks are labeled A′ and B′ in Figure 1a and appear at 189 and 136 K, respectively. In addition to *R*_1_, spin-lock NMR reveals a third, rather prominent rate peak (C′) that is shifted toward much higher temperature (267 K) revealing slower hopping processes than those sensed by peaks A′ and B′.

**Figure 1:**
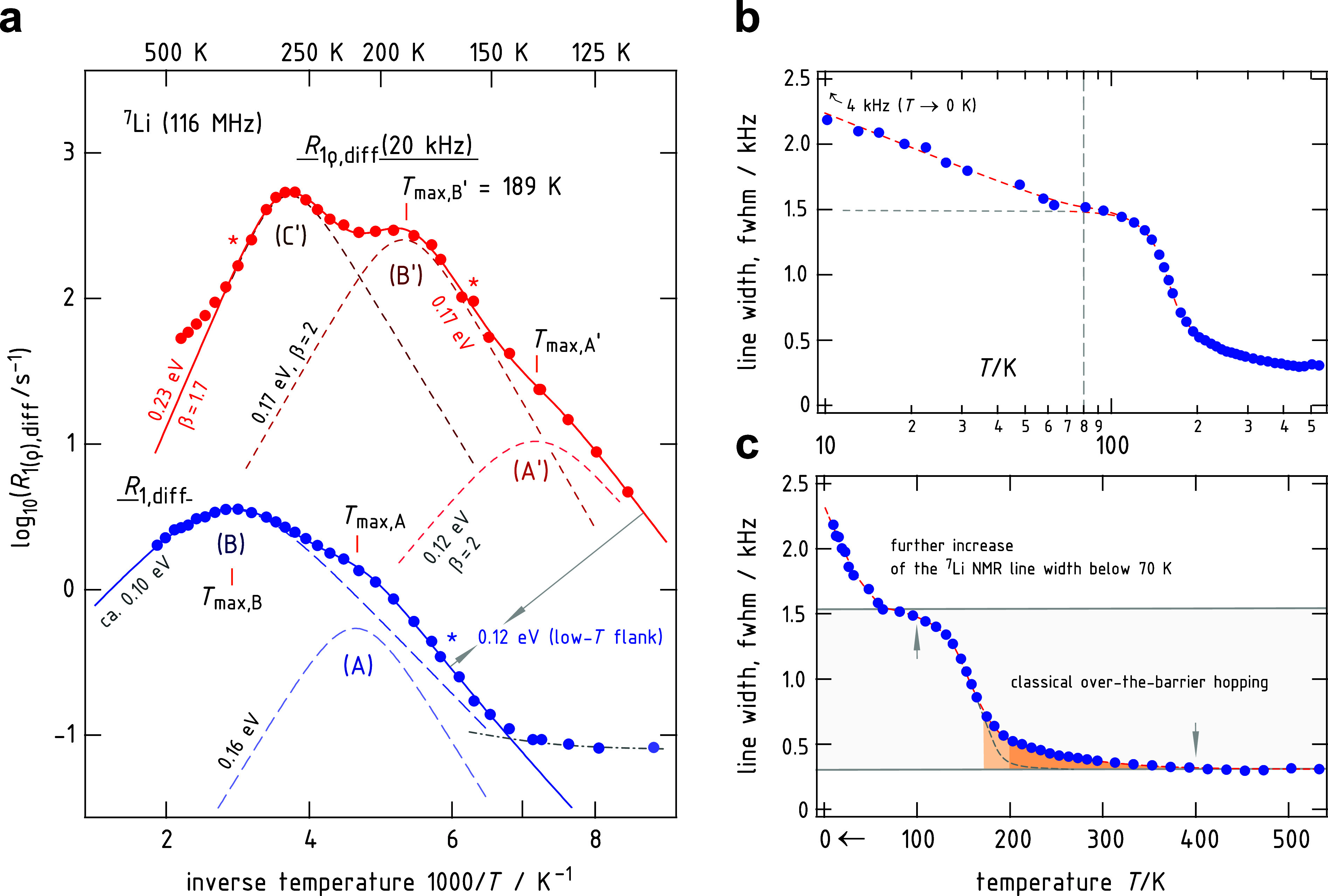
(a) ^7^Li NMR SLR rates (ω_0_/2π = 116 MHz) of polycrystalline LTPS recorded as a function of temperature and analyzed in the frame of an Arrhenius representation, that is, plotting log_10_(*R*_1(ρ)_) vs the inverse temperature 1/*T*. Solid lines show BPP approximations either using two or three individual rate peaks to parametrize the overall ^7^Li NMR response in both the laboratory (*R*_1_, ω_0_/2π = 116 MHz) and rotating frame of reference (*R*_1ρ_, ω_1_/2π = 20 kHz). At low *T*, the rate peak passes into a local regime where it seems to be rather independent of *T*. From the flanks, each marked with an asterisk, we can determine the activation energies for some of the distinct diffusion processes in LTPS. (b,c) ^7^Li NMR line widths of the central transition. (b) Shows the same data as in (c) but using a semilogarithmic plot. See text for further explanation.

To analyze the full dynamic picture in LTPS seen by nuclear spin relaxation, we used a combination of two (*R*_1_) and three (*R*_1ρ_) BPP-type (Bloembergen, Purcell, Pound)^98,108,109^ spectral density terms *J*_1(ρ),i_(ω_0(1)_,τ) (*i* = A, B or A′, B′, C′) to approximate the behavior of *R*_1(ρ)_(1/*T*) over the whole temperature range covered in Figure 1a. The rates at very high (*R*_1ρ_) and very low *T* (*R*_1_), which deviate from the steep decrease expected for BPP-type behavior have been excluded from this analysis. While the former suggests an additional relaxation peak that is not resolved here (and has no effect on the measured rates and the analysis), the latter are included in our analysis when considering measurements down to much lower temperatures (see below). Each *J*_*i*_ contains a single Lorentzian-shaped term according to a BPP analysis allowing for an asymmetric shape of the peak,^98,110,111^ thus, we have *J*_1,*i*_(ω_0_, *T*) = *C* × τ_c_/(1 + (ω_0_τ_c_)^β^) for *R*_1_ and *J*_ρ,*i*_(ω_1_, *T*) = *C*_ρ_ × τ_c_/(1 + (2ω_1_τ_c_)^β^) for *R*_1ρ_. *J*_1(ρ)_, being proportional to *R*_1(ρ)_, is the Fourier transform of the underlying motional correlation function *G*(*t*′). *C*_(ρ)_ denotes the respective coupling constant. The asymmetry parameter β­(∈ R ) ∈ ]­1, 2] describes the frequency dependence of *R*_1(ρ)_ on the low-*T* side of the respective rate peak. β = 2 is obtained in the frame of the isotropic BPP model developed for uncorrelated 3D diffusion^108,109^ on the basis of an exponential function *G*(*t*′). The motional correlation rate τ_c_^–1^ is here assumed to follow Arrhenius behavior, τ_c_^–1^ = τ_0_^–1^ exp­(−*E*_a_/(*k*_B_ *T*)) where k_B_ denotes Boltzmann’s constant and τ_0_^–1^ the pre-exponential factor. The latter is the attempt frequency of the jumping ion, which is expected to be in the order of phonon frequencies (10^12^ to 10^14^ s^–1^).^112,113^ *E*_a_ denotes the activation energy, which is, in the case of 3D diffusion, identical with *E*_a,hT_ available from a line fit of the high-*T* side of *R*_1(ρ)_(1/*T*).^111^ While for β = 2 a symmetric peak is obtained, β < 2 leads to a subquadratic frequency dependence of *R*_1(ρ)_ and to an asymmetric peak with *E*_a,lT_, corresponding to the low-*T* flank, obeying the relation *E*_a,lT_ = (β – 1)*E*_a,hT_. The solid lines in Figure 1a show the overall fits for *R*_1_(1/*T*) and *R*_1ρ_(1/*T*); the dashed lines represent the individual rate peaks. Values in eV denote the activation energies of the BBP-type fits. The best results, summarized in Table 1 for the individual peaks i, were obtained with β = 2, except for peak C′ (β = 1.7) and D (see below). Choosing β = 1.7 resulted in a better overall fit, which also includes peaks A′ and B′. At *T*_max_, the jump rate τ^–1^ ≈ τ_c_^–1^ takes values of 7.3 × 10^8^ s^–1^ (τ^–1^ ∼ ω_0_) and 1.3 × 10^5^ s^–1^ (τ^–1^ ∼ ω_1_), respectively,^96,98^ hence revealing extremely fast ion dynamics at low temperatures.

**Table 1:** Dynamic Parameters as Extracted from the BPP-Type Fits Shown in Figure 1a and from the Low-*T* Peak D Shown in Figure 3b,c^c^

NMR peak, *T*_max_	*E*_a_ (eV)	τ_0_^–1^ (s^–1^)	β	*C* (s^–2^)	diffusion process
A (*R*_1_), 214 K	0.16(1)	3.8(1) × 10^12^	2	7.9(9) × 10^8^	process 2 (and 1)
B (*R*_1_), 343 K^a^	0.10(2)	2.1(4) × 10^10^	2	5.2(1) × 10^9^	process 1, inter-ring
A′ (*R*_1ρ_), 136 K	0.12(4)	4.5(9) × 10^9^	2	5.0(9) × 10^6^	process 2, inter-pocket
B′ (*R*_1ρ_), 189 K	0.17(1)	8.4(8) × 10^9^	2	1.3(1) × 10^8^	process 1, inter-ring
C′ (*R*_1ρ_), 267 K	0.23(1)	4.3(5) × 10^9^	1.7	2.5(1) × 10^8^	slow inter-ring^b^
D (*R*_1_), 125 K	0.065(2)	4.0(5) × 10^11^	1.8	9.1(1) × 10^7^	process 3, intrapocket

a Peak B could already be influenced by the diffusion process represented by peak *C*′.

b a slower diffusion pathway that would be seen in *R*_1_ at higher *T* only.

c At least three diffusion processes could be identified by ^7^Li NMR SLR measurements. While *E*_a_ refers to the high-*T* activation energy probing long-range ion dynamics, which is in contrast to *E*_a,lT_ that senses short-range ion dynamics. *C* denotes the coupling strength of the rate peak, determining the magnitude of the peaks in the Arrhenius diagram, *J*_1(ρ)_(ω_0(1)_,*T*) = *C* × τ_c_/(1 + ((2)­ω_0(1)_τ_c_)^β^) ∝ *R*_1(ρ)_, with τ_c_^–1^ = τ_0_^–1^ exp­(−*E*_a_/(*k*_B_ *T*)). τ_c_^–1^ is equal to the jump rate τ^–1^ within a factor of 2.

We notice that activation energies range from 0.10 to 0.23 eV for the individual diffusion processes. For comparison, long-range ion transport probed by PFG NMR yields an activation energy of 0.25 eV, while conductivity spectroscopy and AIMD simulation resulted in 0.28 and 0.20 eV, respectively.^55^ The latter value agrees well with the activation energy extracted for peak B′ which includes the rate-limiting step for long-range diffusion, that is, jumping between the ring-like structures separated by a distance a of 4–4.5 Å. The corresponding microscopic diffusion coefficient D_SLR_ turns out to be 8.5 × 10–15 m^2^ s^–1^ at 189 K, calculated via the Einstein–Smoluchowski equation *D* = *a* ^2^ × (6τ)^−1^. If we extrapolate the PFG NMR data (360 to 250 K) from Di Stefano et al. down to 180 K,^55^ an overall macroscopic diffusion coefficient, collecting not only the jump processes seen by peak B′, of approximately 4 × 10^–14^ m^2^ s^–1^ is suggested. Considering the differences in how the two methods probe ion dynamics, including possible effects from lattice expansion and the expected variations due to correlated motion between *D*_SLR_ and *D*_PFG_, the agreement is surprisingly good. The same agreement is found if *D*_SLR_(343 K) = 2.4 × 10^–11^ m^2^ s^–1^ of the corresponding peak B is compared with results from PFG NMR (ca. 4 × 10^–11^ m^2^ s^–1^)·^55^

While for the spin-lock experiments relatively low prefactors are obtained (see Table 1), for peak A the factor τ_0_^–1^ adopts values in the order of typical phonon frequencies. Peak B appears to be rather broad, resulting in both a low activation energy and a low prefactor. This peak may already be influenced by several diffusion processes, which are nonetheless distinguishable in spin-lock NMR, as it is sensitive to motional correlation rates in the kHz range (see peaks B′ and C′).

Considering the crystal structure of LTPS, see Figure 2, we can try to assign the individual relaxation processes seen in NMR to specific diffusion pathways and hopping processes. As has been shown by molecular dynamics simulations,^55^ Li diffuses along the Ti–P–S framework, forming segmented “rings” around the Ti sites in the *ab*-plane. These rings are also connected along the *c*-direction, creating a 3D diffusion network. The simulations have shown that at lower temperatures (600 K), regions of high Li residence probability appear as “pockets”, with three pockets forming one ring. Li ions jump between pockets either within the same ring (intraring jumps, pathway 2 in Figure 2) or between the rings (inter-ring jumps; pathway 1 in Figure 2). The intraring jumps proceed on a shorter length scale, while inter-ring jumps, occurring at a lower rate, are the limiting steps for macroscopic Li diffusion, that is, long-range ionic transport. Jumps restricted to the area of a single pocket are denoted as jump process 3 in Figure 2. In Table 1, we tentatively assign the NMR rates peaks to the diffusion processes shown in Figure 2.

**Figure 2:**
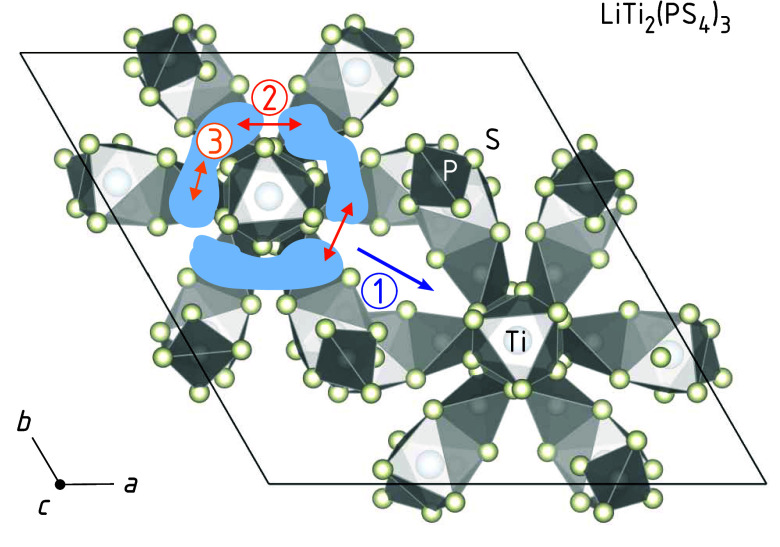
Crystal structure of LiTi_2_(PS_4_)_3_ highlighting the ring-like structure with the Li pockets. Li ions can move within one of the three pockets shown (3), jump between them (2) and jump between the rings (1). The latter jump process is the rate-determining step for long-range transport. It will, most likely, involve interstitial sites the diffusing ions visit.

It has been shown that the pockets with high Li residence probability do not entail typical crystallographic sites, instead these areas are much larger and take the form of “potato”-shaped regions, even at room temperature as obtained from AIMD simulations.^55^ In these potato-shaped pockets, Li occupies highly distorted tetrahedral-like sites with coordination numbers between 3 and 4, that is, different from the typical regular tetraehadral sites in other Li ion conductors. We assume that these intrapocket Li sites are connected by rather low activation energies. As suggested by AIMD,^55^ the Li ions inside the pockets are exposed to soft vibrational modes. Vibrational modes with low frequencies lead to a low attempt frequencies, which might be in line with the prefactors shown in Table 1. On the other hand, such soft modes stabilize the Li sites from an entropic point of view because softer modes lead to a higher vibrational entropy.^55^

Identifying any ultrarapid Li^+^ jump processes, that is, any spatially localized intrapocket Li^+^ jump processes, might require NMR measurements conducted at much lower temperatures as is shown in Figure 3. For this purpose, we carried out ^7^Li NMR SLR rate measurements and line shape measurements (Figures 1b and 3a) down to temperatures as low as 10.2 K (Figure 3b). In Figure 1b,c the full width at half-maximum of the ^7^Li NMR central transition is shown down to such low temperatures. While the decay step observed at temperatures above 100 K is still attributed to jumps between the pockets and the rings, the continuous increase in line width at lower temperatures (*T* < 80 K, Figure 1b) may be related to much faster jump processes within certain sites inside the pockets, connected by very low activation barriers. Indeed, the corresponding ^7^Li NMR SLR rates (see Figure 3b) yield quantitative insights to determine the activation energy behind. As is already seen in Figure 1a, *R*_1_ enters a local plateau at 125 K. Complementary measurements shown in Figure 3b (and in Figure 3c) reveal that this plateau passes into a further linear decay of *R*_1_ if analyzed in an Arrhenius plot. The slope of the corresponding low-*T* flank yields an activation energy of only 39 meV (dashed line in Figure 3c), which we identify as an average value of barriers the Li^+^ ions are subjected to within the pockets (jump process 3 in Figure 2). Analyzing this peak in the frame of the BPP model, we obtain the values shown in Table 1; the higher activation energy (65 meV) is a result of an extrapolation of the low-*T* rates toward higher *T*, see the dotted lines in Figure 3c. The latter rates were approximated with a power law of the form *R*_1_ ∼ *T* ^κ^ (see below and the inset in Figure 3c). The location of the whole *R*_1_(1/*T*) peak (D) on the reciprocal temperature axis indicates that the average residence time of a Li^+^ ion within a pocket is just 1.3 ns (∼ω_0_^–1^) at 125 K highlighting the extremely high local mobility of the charge carriers.

**Figure 3:**
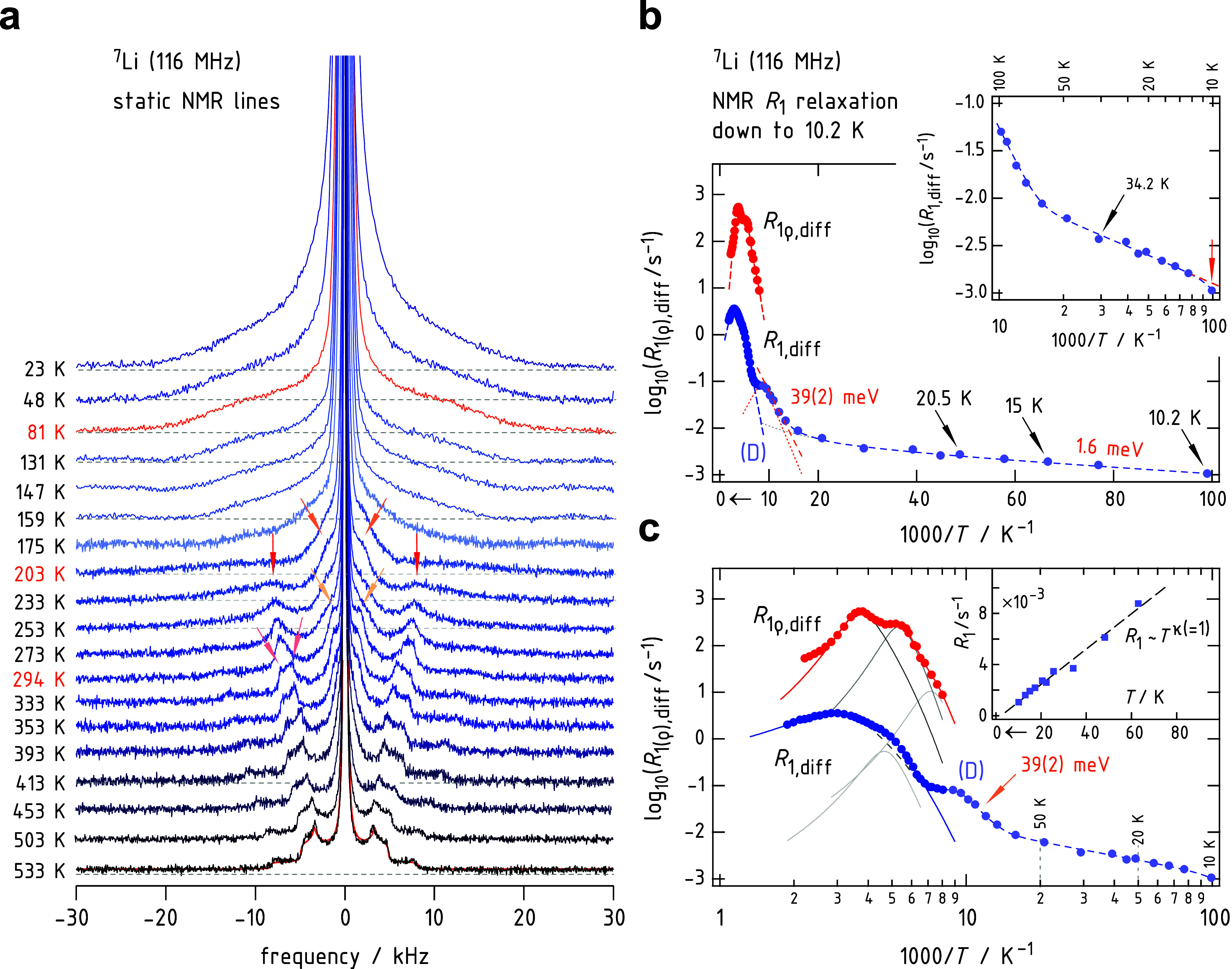
(a) ^7^Li (*I* = 3/2) NMR spectra recorded at the indicated temperatures, optimized to best visualize changes in the quadrupole powder pattern. Below 23 K, the broadened quadrupole pattern remains unchanged, while the central line continues to widen, reaching 2.25 kHz at 10 K. The line recorded at 81 K marks the end of the first narrowing step, see the motional narrowing curve in Figure 1b,c. Arrows indicate the emergence of inner and outer 90° satellite singularities. The red solid line behind the spectrum recorded at 533 K represents a powder pattern simulation with *C*_q_ = 16.6 kHz and η_q_ = 0.19 (see text). (b) Arrhenius representation of the ^7^Li NMR rates measured down to 10.2 K. The local rate peak at 125 K (labeled D), with a low-*T* flank corresponding to an activation energy of 39 meV, transitions into linear decrease of *R*_1_, which exhibits only a very weak temperature dependence (1.6 meV). The inset presents the same data on a temperature scale from 100 K down to 10 K, enhancing the visibility of the slope change (39 meV vs 1.6 meV). (c) The same Arrhenius plot as in (b), but with the reciprocal temperature axis displayed on a logarithmic scale. The inset shows the linear temperature dependence of *R*_1_(*T*) for *T* < 80 K.

The multiple diffusion pathways and hopping processes observed in ^7^Li NMR SLR are also reflected in the changes of the electric quadrupole pattern in the corresponding ^7^Li NMR spectra shown in Figure 3a. When spin-3/2 ^7^Li nuclei experience nonzero electric field gradients (EFGs) at their sites, the resulting modification of the Zeeman levels leads to characteristic quadrupolar powder patterns, depending on the strength and symmetry of the coupling. If the Li^+^ hopping processes between multiple sites in LTPS with different EFGs are sequentially activated with increasing temperature, a corresponding stepwise averaging of the quadrupolar electric couplings is expected. This progressive change in the overall powder pattern is clearly visible in Figure 3a.

Starting from a broad and less structured quadrupole pattern at 23 K (and below), the satellite intensity evolves with increasing temperature. The broad pattern at very low temperature most likely reflects a distribution of EFGs in the limit of very slow diffusion. By 81 K, it adopts the shape of a dipolarly broadened spin-3/2 pattern (intrapocket jumps). At this temperature, the main motional narrowing process starts, see Figure 1b. The corresponding averaging process appears to be completed at 203 K (interpocket and inter-ring jumps). With further temperature increase, an outer pair of satellites emerges and eventually splits at even higher temperatures (see arrows). In parallel, a pair of inner satellites (indicated by arrows) becomes visible at 203 K (further inter-ring jumps); this new pattern is fully developed at 294 or 333 K, and disappears above 400 K. This latter change indicates significantly slower Li^+^ exchange processes (peak C′) than those associated with the relaxation rate peaks appearing at lower *T*. Ultimately, a fully averaged powder pattern is observed at *T* > 500 K, which can be accurately simulated (Figure 3a) using a single set of quadrupole parameters (coupling constant *C*_q_ = 16.61 kHz, asymmetry parameter η_q_ = 0.19). The shift of the already developed 90° singularities (*T* > 450 K) on the frequency scale is presumably due to a slight expansion of the LTPS lattice. The change in the coupling constant is rather small; any influence on ion dynamics might be reflected in the SLR NMR rates recorded at the highest temperatures. Altogether, the sequential evolution of the ^7^Li NMR spectra aligns perfectly with the presence of multiple hopping processes in LTPS, which are activated step by step with increasing temperature—a situation that is very similar to that in Li_12_Si_7_.^102,114^

Returning to the extremely rapid Li^+^ exchange processes observed at temperatures as low as 125 K, we considered whether, in addition to classical over-the-barrier hopping, other dynamic mechanisms might contribute to the overall Li^+^ dynamics in LTPS. Notably, at temperatures below approximately 60 K, the ^7^Li NMR rate *R*_1_ transitions into a regime with very weak temperature dependence. From Figure 3b, we determine an activation energy as low as 1.6 meV for this regime, which persists down to 12 K; the rate recorded at 10.2 K seems to slightly deviate from this trend, see also the inset in Figure 3b. Importantly, within this same temperature range, we observe a shallow yet continuous increase in the ^7^Li NMR line width (Figure 1b,c). Due to the relatively large pockets providing several sites for the Li^+^ ions connected through low-energy barriers, the likelihood of Li^+^ ions undergoing quantum tunneling appears plausible.^115^ Even at very low temperatures, such processes would prevent the complete freezing of ion dynamics, resulting in a continuous increase in NMR line width down to 10 K. For example, assuming a Gaussian-type barrier width of 1 Å and a height of 40 meV, the tunneling probability *T*_P_ for ^7^Li at 20 K would be approximately 0.002. Reducing the barrier height to 20 meV and the width to 0.8 Å increases *T*_P_ to 0.05, and even at 8 K, *T*_P_ remains around 0.02. These rough estimates demonstrate that quantum tunneling of heavier particles than H or D is far from negligible. However, in contrast to studies on H^+^ and D^+^ quantum tunneling,^115,116^ the rate *R*_1_ still shows a weak but measurable temperature dependence in the ultralow-*T* regime (1.6 meV). Therefore, we believe that besides quantum tunneling also classical over-the-barrier hopping processes might contribute to the nuclear spin-relaxation behavior, at least if we consider temperatures ranging from 60 to 10 K.

Dynamic processes of other charge carriers such as electrons or the direct coupling of Li spins to conduction electrons might be another source of low-temperature relaxation. Indeed, we found *R*_1_ ∼ *T* ^κ^ with κ = 1 (see the inset of Figure 3c) in agreement with nuclear spin-relaxation determined by conduction electrons. For comparison, the electronic conductivity of LTPS containing Ti^4+^ cations has been reported to be 8.2 × 10^–8^ S cm^–1^, which is low but non-negligible especially if we consider phenomena at extremely low temperatures. Hence, this additional source of nuclear spin relaxation may obscure the temperature-invariant relaxation mechanism^115^ anticipated for quantum tunneling, as suggested by NMR line shape measurements.

## 4 Conclusions

LTPS has proven to be a fascinating model system for uncovering details of ultrafast Li^+^ hopping processes within a structure that enables multiple, sequentially activated hopping mechanisms. Above 60 K, the ^7^Li NMR SLR rate *R*_1_ is purely diffusion-induced, eliminating the need for corrections due to nondiffusive background effects. This allowed us to precisely determine a sequence of activation energies governing ultrafast Li^+^ dynamics in LTPS, spanning from 0.10 to 0.23 eV and characterizing both intra- and inter-ring exchange processes.

The stepwise activation of distinct hopping mechanisms is directly reflected in the sequential changes of the corresponding electric quadrupolar powder pattern in the ^7^Li NMR spectra. At temperatures extending into the cryogenic regime, we cannot rule out a contribution from ^7^Li (and ^6^Li) quantum tunneling to the overall *R*_1_ rate, which exhibits only a very weak temperature dependence. This assumption is further supported by the absence of a classical rigid-lattice regime in the ^7^Li NMR line width at the lowest temperature. Instead, we observe an anomalous increase in line width down to 10.2 K.

## Supplementary Material



## Notes

Brief note on the shape of the NMR transients (PDF)
