# Supplementary material for: Unraveling Ultrafast Li-Ion Dynamics in the Solid Electrolyte LiTi2(PS4)3 by NMR down to Cryogenic Temperatures
Source: J Am Chem Soc. 2025 Jun 2;147(23):20023–32. doi: 10.1021/jacs.5c05253 (PMC12164270; doi:10.1021/jacs.5c05253)
Supplement: Supplementary file 1 [file ja5c05253_si_001.pdf]

Unraveling Ultrafast Li-Ion Dynamics in the Solid Electrolyte $LiTi_2(PS_4)_3$ by NMR Down to Cryogenic Temperatures

Denise Tapler,[1] Bernhard Gadermaier,[1] Jonas Spychala,[1] Florian Stainer,[1] Annika Marko,[1] Jana Königsreiter,[1] Katharina Hogrefe,[1] Paul Heitjans,[2] and H. Martin R. Wilkening[1]*

[1]*Graz University of Technology, Institute of Chemistry and Technology of Materials (NAWI Graz), Stremayrgasse 9, 8010 Graz, Austria*
[2]*Leibniz Universität Hannover, Institute of Physical Chemistry and Electrochemistry, Callinstraße 3-3a, 30167 Hannover, Germany.*

* corresponding author: wilkening@tugraz.at

Shape of the NMR transients

As Wimperis *et al.* [1] demonstrated for spin-3/2 nuclei subjected to non-vanishing electric field gradients and undergoing diffusion-driven spin-lattice relaxation, the recovery of longitudinal magnetization following excitation by strong radio-frequency pulses is expected to exhibit a bi-exponential time dependence. This work builds on earlier considerations by Hubbard [2]. A concise summary is also provided in ref. [3]. Under specific conditions, the two spin-lattice relaxation rates can differ by a factor of less than 10, depending on the precise nature of the system. For instance, a single-exponential time dependence is expected when the motional correlation function *J* is single-exponential, and a uniform spin temperature has been established, as typically occurs at sufficiently high temperatures [4, 5]. However, in conventional relaxation rate measurements, resolving these two distinct rates can be challenging and depends on the temperature limit and the spin temperature of the system. Confounding factors, such as additional relaxation mechanisms (e.g., dipolar spin interactions) and the presence of multiple coupled spin reservoirs, can obscure the idealized bi-exponential behavior expected for a single reservoir, even in the presence of moderate quadrupolar interactions, rapid particle diffusion, and in the low-temperature limit.

Here, the combined effects of magnetic dipolar and electric quadrupolar interactions, along with multiple diffusion mechanisms in $LiTi_2(PS_4)_3$, give rise to transients (comprising up to 30 data points) that are well captured by mildly stretched exponential functions, with stretching exponents ranging from 0.8 to 1.0. We found no evidence supporting a bi-exponential fit to the recorded transients. Across the investigated temperature range, the stretching factors cluster around 0.9, indicating that the overall relaxation behavior of this complex system is close to single-exponential.

References

[1] S. Wimperis, G. E. Rudman, K. E. Johnston *J. Phys. Chem. C* **128** (2024) 5453, and references therein, *e.g.*, those of A. Abragam, P. S. Hubbard, see [2], and H.-W. Spiess.
[2] P. S. Hubbard, J. Phys. Chem. **53** (1970) 985.
[3] M. Wilkening, *Ultralangsame Ionenbewegungen in Festkörpern*, Logos Verlag, Berlin, 2005.
[4] D. Wolf, *Spin Temperature and Nuclear Spin Relaxation in Matter: Basic Principles and Applications*, Clarendon Press, Oxford, 1969.
[5] K. D. Becker H. Hamann, N. Kozubek, H. Richtering, Ber. Bunsenges. Phys. Chem. **79** (1975) 1124.
